# Albendazole-induced liver injury: a case report

**Published:** 2013-06-30

**Authors:** David Ríos, Juan C Restrepo

**Affiliations:** a Facultad de Medicina, Universidad de Antioquia; Grupo de Gastrohepatologia, Universidad de Antioquia - Hospital Pablo Tobón Uribe. Medellín, Colombia. E-mail: davidriosp90@gmail.com; b Universidad de Antioquia-Hospital Pablo Tobón Uribe, Grupo de Gastrohepatologia, Grupo de Gastrohepatologia, Universidad de Antioquia - Hospital Pablo Tobón Uribe. Medellín, Colombia. E-mail: jcrestrepo@hptu.org.co

**Keywords:** Hepatitis, albendazole, drug-induced liver injury DILI

## Abstract

We report a case of a 47-year-old male, who was referred to the clinical hepatology services at Pablo Tobón Uribe Hospital for evaluation of a jaundice syndrome. After undergoing several exams, we diagnosed hepatic hydatidosis and the patient was treated with albendazole; however, after five months of uninterrupted treatment the patient again consulted and his liver test showed marked hepatocellular damage. This time, the patient was diagnosed with drug-induced liver injury due to albendazole, based on information from the clinical record, history of drug consumption, clinical and laboratory tests improved after discontinuing the medication and after discarding other possible causes; this diagnosis was supported by the CIOMS/RUCAM scale, which showed a "likely" correlation between hepatocellular damage and drug toxicity etiology.

## Abstract (es)

Presentamos el caso de un paciente masculino de 47 años de edad, quien fue remitido al servicio de hepatología clínica del Hospital Pablo Tobón Uribe para el estudio de un síndrome ictérico. Tras realizársele varios análisis, se le diagnosticó hidatidosis hepática y recibió albendazol como terapia, sin embargo, después de cinco meses de tratamiento ininterrumpido consultó nuevamente y su perfil hepático mostró marcado daño hepatocelular. Se le diagnosticó entonces una hepatitis toxica inducida por albendazol basados en la información de la historia clínica, el antecedente de consumo del fármaco, la mejoría clínica y en las pruebas de laboratorio tras suspender el medicamento y después de descartar las otras causas posibles; este diagnóstico fue respaldado por la escala CIOMS/RUCAM, que mostró una correlación probable entre el daño hepatocelular y la etiología toxica farmacológica.

## Introduction

Albendazole is the medication of choice to treat multiple diseases caused by parasites from the nematode and cestode types, among which there are alveolar echinococcosis and neurocysticercosis^1^ ^,^ ^2^. Additionally, it has been successfully implemented as alternative therapy in other parasitosis, without generating clinically relevant adverse effects^3^ ^,^ ^4^. The drug binds to the β-tubulin of the parasitic cell and impedes microtubule polymerization, which interferes with the mitototic spindle formation and the glucose uptake; it also inhibits mitochondrial fumarate reductase, which alters oxidative phosphorylation and the parasite's energy metabolism, this produces the cell's immobility, spastic paralysis, and - finally - death^5^ ^,^ ^6^.

The secondary effects of the medication are usually mild; reports have been made of constipation, abdominal pain, nausea, drowsiness, dizziness, pruritus, transient hair loss, anorexia, and mild aminotransferase increase^3^ ^,^ ^5^ ^,^ ^7^. Significant adverse reactions compromising the liver and bile ducts are rare, in up to 15.6% of the cases elevation of hepatic enzymes has been reported which decreases with the temporary withdrawal of the medication without generating any comorbidity^7^. To our knowledge, only in two cases have the elevation of transaminases and the accompanying symptomatology obligated the definite suspension of the treatment^8^ ^,^ ^9^.

International specific or absolute parameters have not been established to identify drug-induced toxic hepatitis. Currently, the most accepted diagnostic criteria correspond to the semi-qualitative CIOMS/RUCAM scale^10^ to assess the drug/toxic hepatopathy causality developed in 1990. This method has demonstrated reliability and superiority against other methods published afterward^11^ and it was implemented in this case.

Hereinafter, we describe the case of a patient from our hepatology services, which was diagnosed with toxic hepatitis induced by albendazole.

## Case description

This case included a 47-year-old male patient without antecedents of importance, who consulted due to a two-month clinical condition consisting of fever, generalized jaundice, and pain in the right abdomen associated to paresthesia on the ipsilateral lower limb.

The patient was forwarded to the city of Medellín for evaluation in the clinical hepatology services at Pablo Tobón Uribe Hospital. Upon admission, hepatic profile was taken (results are shown in Table 1) as well as hepatotropic virus serology, which was negative. Thereafter, carcino-embryonic antigen and alpha-fetoprotein were measured and found within normality range.

**Table 1:**
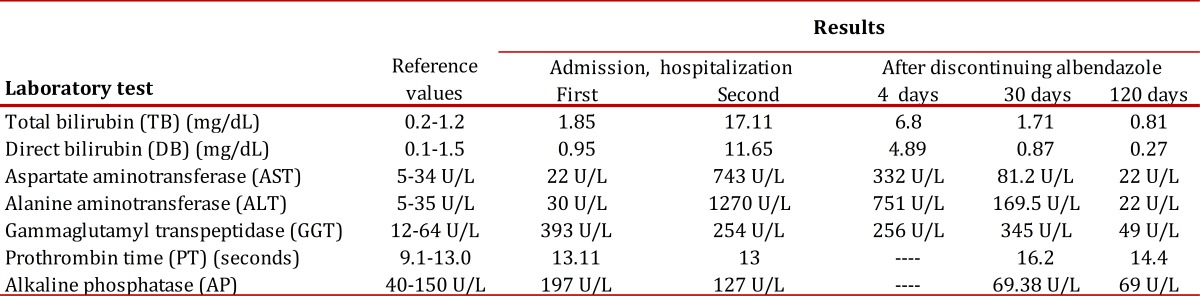
Behavior of the hepatic profile over time, from the first hospitalization to four months after discontinuing albendazole

Magnetic resonance revealed two cystic lesions; the first was located between the middle hepatic vein and the right hepatic vein, with a diameter of 6 x 6 cm, with sub-capsular enhancement, without septa or nodules; the second lesion occupied the whole right lobe of liver with multiple enhancing septa. No ischemic liver damage was found and the bile duct was found normal. According to these findings, hepatic hydatidosis was diagnosed and treatment was begun with 600 mg albendazole every 24 hrs.

After five months of uninterrupted treatment, the patient was again admitted and hospitalized because of jaundice syndrome. Physical exam found mild hepatomegaly and tenderness in the right hypochondrium, icterus in mucosa and dysesthesias in lower right limb; the patient also reported coluria with one-week evolution and denied consumption of other medications or nutritional supplements after discharge.

It was decided to discontinue albendazole and maintain continuous monitoring of the patient's hepatic function. Again, hepatropic virus serology, carcino-embryonic antigen, and alpha-fetoprotein were negative; additionally, a new magnetic resonance showed a mild decrease of cysts.

After four days of hospitalization improved hyperbilirubinemia was noted along with decreased transaminases (Table 1); albendazole-induced liver damage was diagnosed and after establishing progressive clinical and para-clinical improvement, the patient was discharged.

The patient attended the out-patient consultation at Pablo Tobón Uribe Hospital 30 days later; bringing laboratory exams that revealed mild hyperbilirubinemia and decreased transaminases. Three months later, the patient was asymptomatic and his hepatic profile was normal (Table 1).

## Discussion

Drug-induced liver injury (DILI) is a disease of variable clinical presentation, with a spectrum that goes from the lack of symptoms to acute liver failure and even chronic liver disease. It is an under-registered condition and whose diagnosis is of exclusion; hence, it is based on the clinical history, antecedents of drug intake, evaluation of para-clinical exams, and exclusion of other possible causes that mask the toxicity^12^.

In this case, no antecedents of importance was present; hydatidosis was the indication for albendazole use, a drug that has been effectively implemented without clinically significant adverse effects on the liver in similar cases^13^ ^-^ ^15^, which makes it quite unlikely that the inflammatory reaction caused by the parasite's death becomes responsible for hepatocellular damage.

The biochemical profile during the first hospitalization evidenced cholestasis; during the second admission and after five months of anti-parasite treatment the damage pattern shifted to hepatocellular, with marked increase of aminotransferases that lessened progressively with the withdrawal of albendazole until normalizing at four months without need to undertake an additional conduct. Between both hospitalizations there was no therapeutic intervention different from the anti-parasite prescription.

Imaging aids permitted discarding ischemic liver damage; serology for micro-organisms that induce acute and chronic liver lesion was negative, and the differential diagnosis of liver cystadenocarcinoma was discarded with the second magnetic resonance, which showed slight decrease in the size of cysts.

The CIOMS/RUCAM scale was used to objectively evaluate the probability of toxic hepatitis; it showed a "likely" correlation with a 5-point score, which confirmed the diagnosis of albendazole-induced toxic hepatitis with a pattern of hepatocellular damage.

Until now, worldwide, two other cases have been reported of toxic hepatitis due to albendazole^8^ ^,^ ^9^; this is the first case documenting sub-acute toxicity and which describes progressive improvement of the hepatic profile over time, after discontinuing the medication as the unique intervention.

## Conclusion

Given that diagnosis of DILI is of exclusion and sub-reporting is common, it is important for medical personnel to suspect it in every patient with hepatic dysfunction and conduct precise characterization of medication intake and risk factors. Additionally, it must be considered that any drug has hepatotoxic potential and that cases of hepatitis due to medications occur in our environment without being diagnosed.
